# Clinical Significance of Disulfidptosis-related Genes and Functional Analysis in Gastric Cancer

**DOI:** 10.7150/jca.91796

**Published:** 2024-01-01

**Authors:** Jianing Yan, Ziyi Fang, Meiqi Shi, Can Tu, Shengke Zhang, Chenglu Jiang, Qier Li, Yongfu Shao

**Affiliations:** 1 Department of Gastroenterology, The First Affiliated Hospital of Ningbo University, Ningbo 315020, China.; 2 Clinical Medical College, Southwest Medical University, Luzhou 646000, China.

**Keywords:** disulfidptosis, gastric cancer, bioinformatics, prognosis, diagnosis

## Abstract

**Background:** Worldwide, gastric cancer (GC) remains intractable due to its poor prognosis and high morbidity and mortality. Disulfidptosis is a novel kind of cell death mediated by abnormal accumulation of intracellular disulphides. The correlation between disulfidptosis and GC is still unknown. Therefore, it is necessary to elucidate the pathogenesis and mechanism of disulfidptosis and GC for clinical diagnosis and intervention.

**Methods:** RNA-sequencing data from several public data portals and clinical samples were collected. We compared the expression levels of four key genes of disulfidptosis, including SLC7A11, SLC3A2, RPN1, and NCKAP1, in GC and selected prognostic genes to build a novel GC prognosis-related nomogram model. The biological functions and immune landscape of the identified prognostic genes were explored.

**Results:** Overexpressed NCKAP1 and SLC7A11 were prognostic disulfidptosis-related genes in GC. We combined these genes and several clinicopathological factors to build a prognostic nomogram model for GC. Meanwhile, the ROC curves showed that NCKAP1 and SLC7A11 were promising biomarkers for GC screening. The biological and cellular functions were focused on actin activities, GTPase and immunoreaction. The tumour immune microenvironment and immune therapy targets were identified. Competing endogenous RNA network was built to explore the downstream regulatory mechanisms. Finally, the elevated NCKAP1 and SLC7A11 expression in GC was validated via qRT-PCR in a cell line and tissue line.

**Conclusion:** In conclusion, NCKAP1 and SLC7A11 are promising prognostic and diagnostic biomarkers for GC that correlate with the activities of actin, energy metabolism of GTPase, immune infiltration and immunotherapy.

## 1. Introduction

Gastric cancer (GC) is a relatively common digestive tract malignancy with the fourth highest incidence and mortality rate worldwide^1^. GC lacks typical symptoms and signs at an early stage, so most patients are diagnosed in the advanced stage with lymph node and distal invasion^2^, ^3^. The detailed molecular biological mechanisms of GC initiation and development are poorly understood. As one of the emerging breakthroughs for cancer therapy, there are still no available and effective targets for immunotherapy^4^. Hence, the prognosis of GC is always poor, with a low five-year survival rate of approximately 20%^5^. It is urgent to explore a satisfactory diagnostic and prognostic tool to guide gastric cancer therapy and improve clinical outcomes.

Disulfidptosis is a novel kind of cell death mediated by abnormal accumulation of intracellular disulphides in which glucose transporter inhibitors induce disulfidptosis in glioma cells and suppress tumour growth, suggesting a potential clinical application and treatment strategy^6^. In addition, it has been shown that SLC7A11, SLC3A2, RPN1, and NCKAP1 are the key genes required for the progression of disulfidptosis^6^. To date, the clinical significance and value of disulfidptosis-related genes are unknown, so it is necessary to assess the roles of these genes in gastric cancer.

Therefore, we developed and validated a prognostic nomogram and combination diagnosis model for disulfidptosis-related genes in this study. We further investigated the competing endogenous RNA (ceRNA) regulatory mechanisms, biological function, immune microenvironment and immunotherapy-related drugs. We aimed to illustrate the clinical value of disulfidptosis-related genes for improving the distal outcomes of GC patients and characterize the immune landscape to provide novel immunotherapy targets for clinical application.

## 2. Materials and methods

### 2.1 Public database retrieval and clinical data acquisition

We completely downloaded the clinical and pathological information of gastric cancer and the RNA sequencing (RNA-seq) data collected by The Cancer Genome Atlas (TCGA) database (https://genome-cancer.ucsc.edu/) and normalized RNA-seq data from the Genotype-Tissue Expression (GTEx) data portal (https://www.gtexportal.org/home/index.html). Two human GC cell lines (SGC-7903 and MGC-803) and the immortal human stomach cell line GES-1 were obtained from the Shanghai Institute of Biochemistry and Cell Biology, Chinese Academy of Sciences, China. Clinical samples such as GC tissues and paired adjacent nontumorous tissues (5 cm away from the edge of the tumour) were collected from 30 patients who received gastrectomy from the Affiliated Hospital of Medical School of Ningbo University, China, between 2022 and 2023. All patients signed informed consent forms, and this study was approved by the Ethics Committee of the Affiliated Hospital of Medical School of Ningbo University (No. KY20220101).

### 2.2 Distinguishingly expressed and prognostic disulfidptosis-related gene identification

We first compared the expression levels of disulfidptosis-related genes in gastric cancer between the TCGA cohort and normal tissues in the GTEx cohort using *t* tests or Wilcoxon rank-sum tests. The associations of the expression level of disulfidptosis-related and Mismatch Repair Gene (MMR) were examined in TCGA cohort 7. The CBio Cancer Genomics Portal (http://cbioportal.org) was used to explore multidimensional alterations in disulfidptosis-related genes in TCGA GC sample**s** 8. The Kaplan-Meier method was used to analyse the GC survival data from this cohort via R software (version 4.2.1) and the R package survival v 3.3.1. Univariate regression and multivariate regression were utilized to assess significant clinical prognostic factors. The results of the multivariable model were shown as forest plots via the forest plot function in R software. The risk score model was constructed by the sum of each prognostic risk factor with the following formula: risk score = expression level of Gene 1 × β_1_ + expression level of Gene 2 × β_2_ + … + expression level of Gene n × β_n_ 9_._ All of the patients in the TCGA cohort were computed via the prognostic performance of the risk score model.

### 2.3 Construction and validation of the disulfidptosis-related prognostic nomogram model

The risk factors in the multivariate regression and risk score model were incorporated into the prognostic nomogram model. The 1-, 3- and 5-year OS overall survival time (OS) prediction nomogram model was established by the R packages survival [3.3.1] and rms [6.3-0] in R software. A calibration curve was obtained, and the line on the diagonal 45-degree line suggested an ideal nomogram 10. Decision curve analysis (DCA) was also performed to assess the clinical net benefit 11.

### 2.4 Assessment of diagnostic values of disulfidptosis-related genes

The TCGA cohort and receiver operating characteristic (ROC) curve analysis were used to evaluate the diagnostic values of disulfidptosis-related genes. Then, the GTEx cohort was added to validate the diagnostic effectiveness. Combination diagnosis was performed to improve the diagnostic values.

### 2.5 Biological function analysis of prognostic disulfidptosis-related genes

The GeneMANIA prediction server is a web portal for gathering interactive genes and drawing biological network integration for gene prioritization 12. The interactive genes were chosen to build a protein‒protein interaction (PPI) network via STRING 11.5, an online database for searching and constructing organism-wide protein association networks 13. KEGG pathway enrichment analysis and gene ontology (GO) classification were performed to explore the biological functions of the PPI network via the R packages “clusterProfiler” and “ggplot2” 14-17. A *p* value < 0.05 represents a statistically significant difference.

### 2.6 Immune infiltration landscape analysis

The correlations between the expression level of prognostic disulfidptosis-related genes and immune cell infiltration were analysed by R packages “GSVA (1.46.0)” and “estimate (1.0.13)” with the default parameters 18. The Deeply Integrated Single-Cell Omics data (DISCO, https://www.immunesinglecell.org/) contained comprehensive collections of single-cell RNA-seq datasets of the tumour microenvironment that was used to detect the purity and immune infiltration of GC 19. TISIDB (http://cis.hku.hk/TISIDB/index.php), an online web portal generating multiple heterogeneous data types, was applied to explore immune system interactions and related drugs 20. Pearson's correlation analysis was performed to determine the association between the expression level of genes and indicators (*P* <0.05).

### 2.7 Construction of competing endogenous RNA regulatory network

MicroRNAs (miRNAs) of disulfidptosis-related genes from four prediction databases, including DIANA-microT 2023 (http://diana.imis.athena-innovation.gr/DianaTools/index.php?r=microT_CDS/index) 21, TarBase v.8 (https://dianalab.e-ce.uth.gr/html/diana/web/index.php?r=tarbasev8) 22, miRDB (http://mirdb.org/miRDB/) 23, and miRWalk (http://mirwalk.umm.uni-heidelberg.de/). Target miRNAs were defined as miRNAs found in all four databases 24. Whereafter, target miRNAs were input into mirDIP database (http://ophid.utoronto.ca/mirDIP/index_confirm.jsp) and the “Bidirectional” mode was used to filter the very high confidence RNAs 25. All of twenty data sources were chosen and three or more of the programs as well as the top 1% of the confidence class genes (Very High) were considered as possible target genes. LncBase V.3 (https://diana.e-ce.uth.gr/lncbasev3) helped us to find the long non-coding RNAs (lncRNAs) targeted to the miRNAs 26. LncRNAs with direct validation and at least 3 experiments validation were deemed as target lncRNAs. The potential correlations between RNA binding proteins and mRNAs were acquired from starBase (https://rnasysu.com/encori/index.php) 27.

### 2.8 RNA isolation and quantitative real-time PCR (qRT-PCR)

All of the RNA was extracted from tissue and plasma using TRIzol reagents and TRIzol LS reagents (Ambion, Carlsbad, CA, USA) in this study. Total RNA was used as a template and reverse transcribed to cDNA using a GoScript Reverse Transcription (RT) System (Promega, Madison, WI, USA) based on the manufacturer's instructions 28. qRT-PCR was applied with GoTaq qPCR Master Mix (Promega) on the basis of the manufacturer's instructions on an Mx3005P Real-Time PCR System (Stratagene, La Jolla, CA, USA) repeated twice. The reaction conditions were as follows: denaturation at 95 °C for 15 s, annealing at 50 °C for 30 s, and extension at 72 °C for 30 s for 40 cycles, followed by extension at 72 °C for 7 mins. All of the primers were synthesized by Sangon Biotech (Shanghai, China) and the sequences of the primers were as follows: NCKAP1: forward, 5'-TCCTAAATACTGACGCTACAGCA-3', reverse, 5'-GCCTCCTTGCATTCTTATGTC-3'. SLC7A11: forward, 5'-TTACCAGCTTTTTTACGAGTCT-3', reverse, 5'-GTGAGCTTGCAAAAGGTTAAGA-3. GAPDH: forward, 5'-ACCCACTCCTCCACCTTTGAC-3', reverse, 5'-TGTTGCTGTAGCCAAATTCGTT-3'. The fold change of targeted genes was standardized via the Δ*C*t method (Δ*C*t = *C*t_gene_ - *C*t_GAPDH_), in which a higher Δ*C*t suggests a lower expression level 29. The ΔΔ*C*t method (ΔΔ*C*t = Δ*C*t_GC cell_ - Δ*C*t_GES-1_) was used to compare expression levels in GC cell lines to calculate relative expression, and a higher 2^-ΔΔ*C*t^ value represents a higher relative expression level 30.

### 2.9 Statistical analysis

Analyses in this study were used R software (version 4.2.1), cytoscape (version 3.8.0) or GraphPad (version 8.02), and their support packages as mentioned before. *P*<0.05 was considered significant.

## 3. Results

### 3.1 Disulfidptosis-related genes were differentially expressed in GC

**Figure 1:**
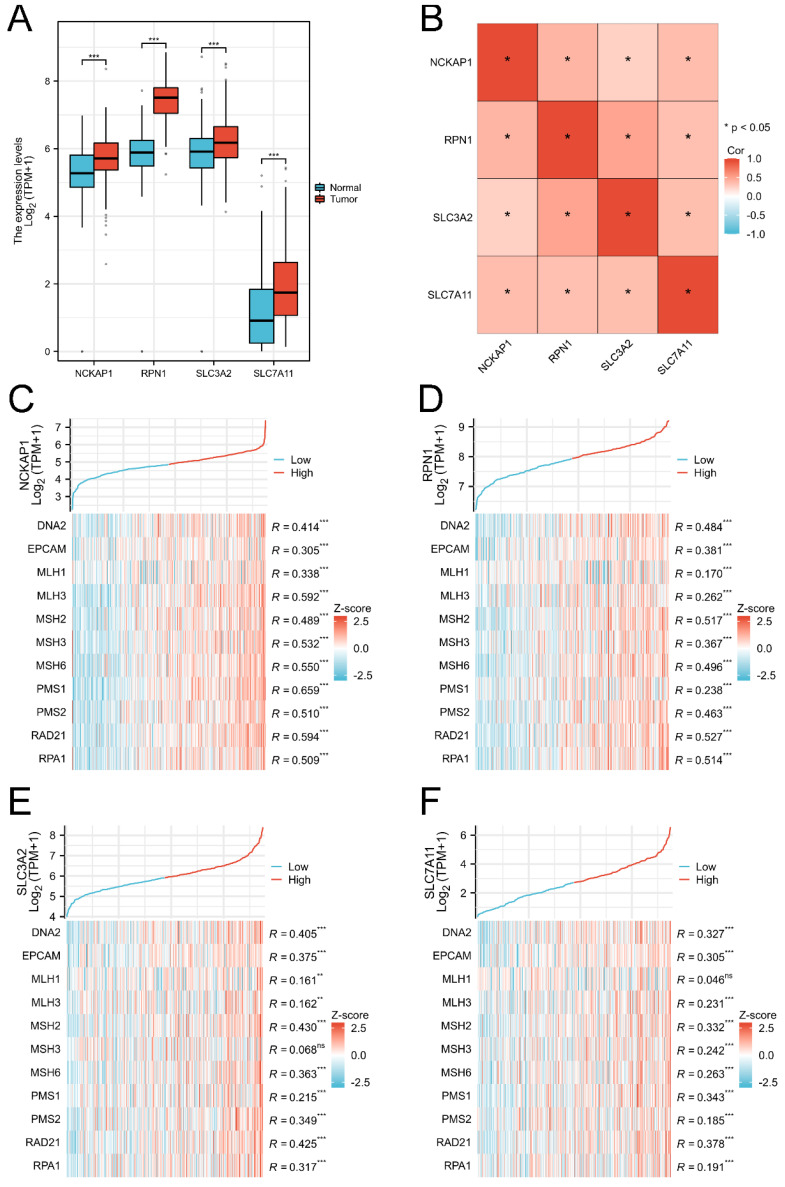
** The expression level of disulfidptosis-related genes in GC.** A: The expression level of NCKAP1, RPN1, SLC3A2, SLC7A11 in GC from TCGA and GTEx cohorts. B: The coexpression analysis of NCKAP1, RPN1, SLC3A2, SLC7A11 in GC from TCGA cohort. C-F: The association between NCKAP1 (C), RPN1 (D), SLC3A2 (E), SLC7A11 (F) and MMR genes in GC from TCGA cohort (*p < 0.05, **p < 0.01, ***p < 0.001).

The RNA-Seq data of 414 GC tissues and 36 para-carcinoma tissues were extracted from the TCGA database, and 174 normal tissues were downloaded from the GTEx database. Our results showed that NCKAP1, RPN1, SLC3A2, and SLC7A11 were consistently overexpressed in GC tissues (*P*<0.001) in Figure (Fig. 1A). Co-expression analysis showed that these genes were significantly elevated, suggesting the internal links of these genes (Fig. 1B). Similarly, the associations between the expression of MMR genes and disulfidptosis-related genes were revealed in Fig 1B-D. The mutation data of these genes in GC and GC subtypes were shown in Supplementary Figure 1 (Fig. S1).

### 3.2 Identification of prognostic disulfidptosis-related genes

**Figure 2:**
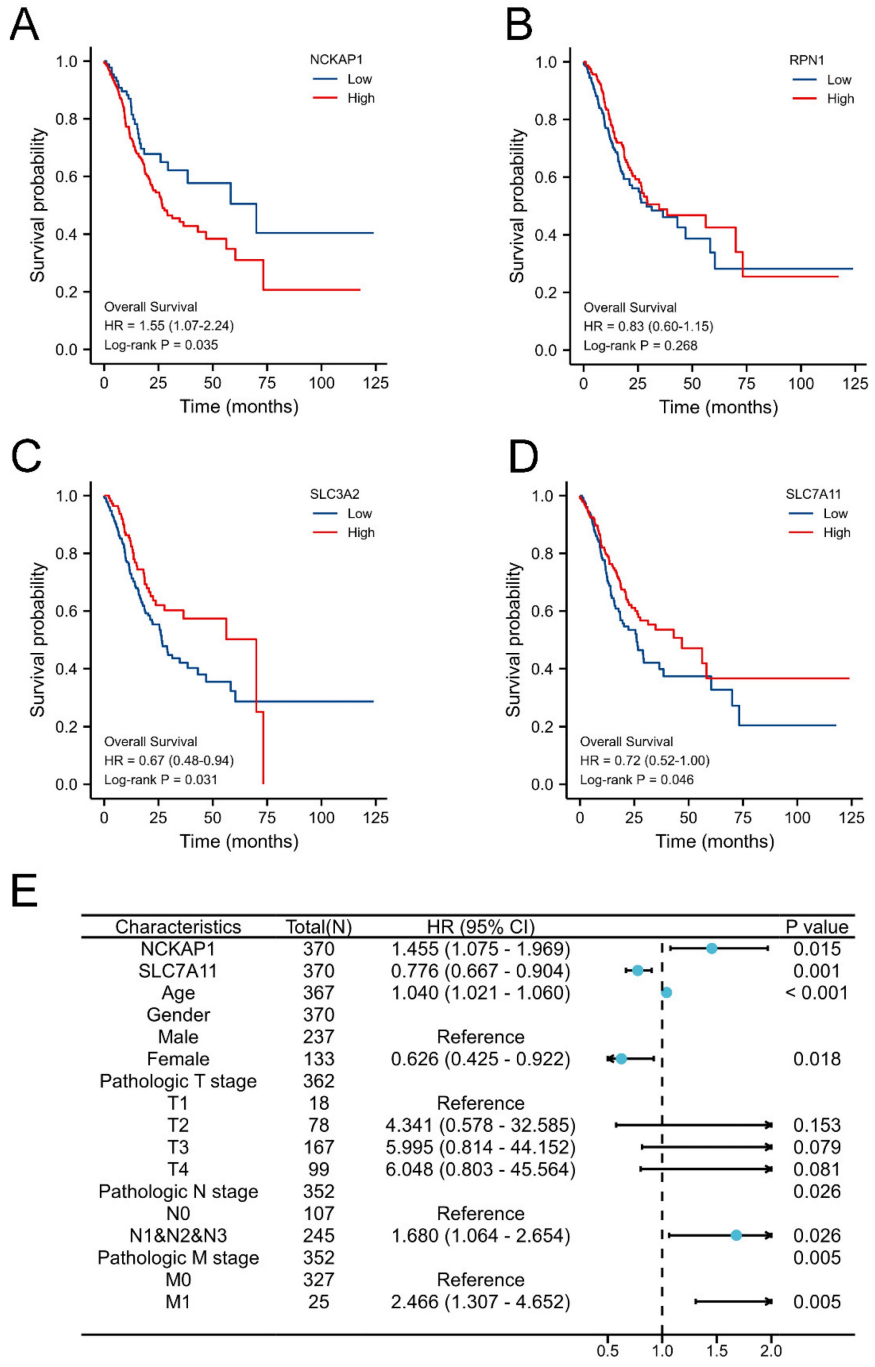
** Identification prognostic values of disulfidptosis-related genes in GC.** A: Overexpression NCKAP1 associated with poor overall survival time in GC. B: The expression of RPN1 did not correlate with the prognosis of GC. C: Overexpression SLC3A2 associated with poor overall survival time in GC. D: Downregulated SLC7A11 associated with poor overall survival time in GC. E: Forest plot of the multivariate COX regression model.

Obviously, the expression levels of NCKAP1, SLC3A2, and SLC7A11 were remarkably associated with OS in GC by Kaplan-Meier methods, as shown in Fig. 2A-D. Combined with common clinicopathologic characteristics (Table S1-2), univariate regression and multivariate COX regression showed that NCKAP1, SLC7A11, age, sex, pathological T stage, pathological N stage, and pathological M stage were independent risk factors for OS (Table 1), which was visualized by a forest plot (Fig. 2E). Then, the prognostic risk score model was constructed according to the multivariate COX regression: Risk score = (1.455*NCKAP1 exp) + (0.776*SLC7A11 exp). The Kaplan-Meier curve showed that the patients with higher risk factors had poor distal overcome (include NCKAP1 and SLC7A11, Fig. S2).

### 3.3 Construction and validation of the prognostic nomogram model

**Figure 3:**
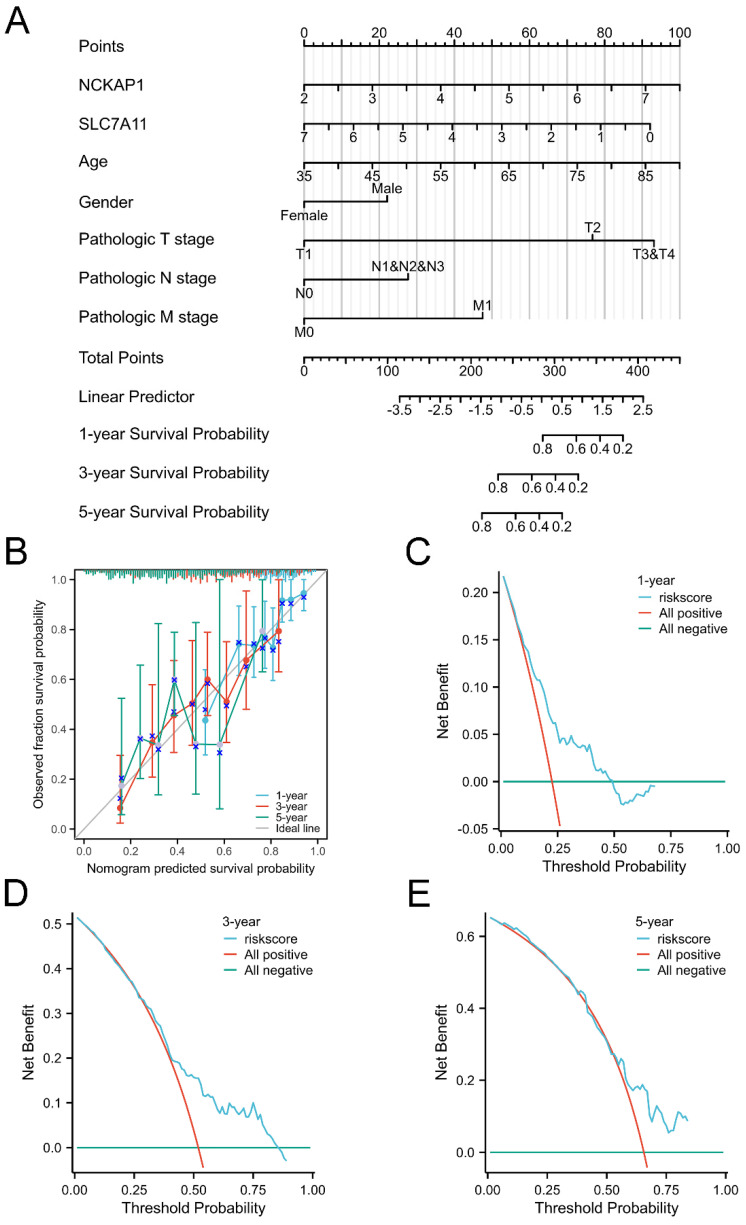
** The overall survival nomogram model and validation.** A: The overall survival nomogram model to predict the 1, 3, and 5, year OS of GC patients. B: The 1, 3, and 5, year calibration plots of the overall survival nomogram model. C-E: The 1, 3, and 5, year DCA curves of the nomogram.

Given the favourable prognostic value of these parameters, we integrated these characteristics and established a prognostic nomogram model to predict the 1-, 3-, and 5-year OS of GC patients, as displayed in Fig. 3A. The C-index of the nomogram model was 0.681 (0.656-0.707). Subsequently, the nomogram calibration plot (Fig. 3B) demonstrated that the model was accurately calibrated to the observed probabilities. The DCA curves in Fig. 3C-E indicated that our nomogram model had satisfactory clinical usefulness.

### 3.4 NCKAP1 and SLC7A11 are promising screening biomarkers of GC

**Figure 4:**
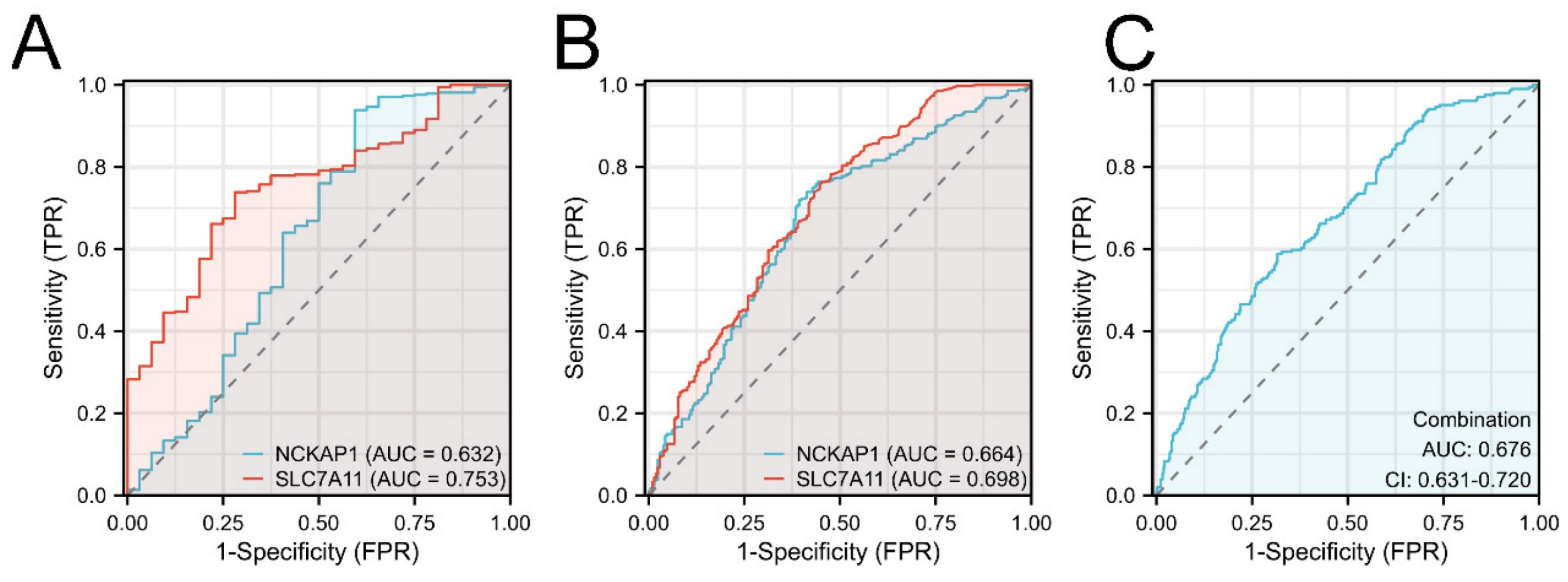
** The ROC curves of NCKAP1 and SLC7A11 of GC.** A: The ROC curves of NCKAP1 and SLC7A11 in TCGA cohort. B: The ROC curves of NCKAP1 and SLC7A11 in TCGA and GTEx cohorts. C: The ROC curve of combination diagnosis of NCKAP1 and SLC7A11 in TCGA and GTEx cohorts.

Furthermore, the diagnostic values of both prognostic genes were detected. We first built the ROC curves of NCKAP1 and SLC7A11 from the TCGA cohort in Fig. 4A. Based on the superior AUC value, we expanded the samples by adding the GTEx cohort for validation. The AUC values in Fig. 4B are 0.664 (NCKAP1) and 0.698 (SLC7A11). Finally, combination diagnosis was performed to improve the diagnostic efficacy, as shown in Fig. 4C (AUC= 0.676, 95% CI= 0.631-0.720).

### 3.5 Biological function analysis of NCKAP1 and SLC7A11

**Figure 5:**
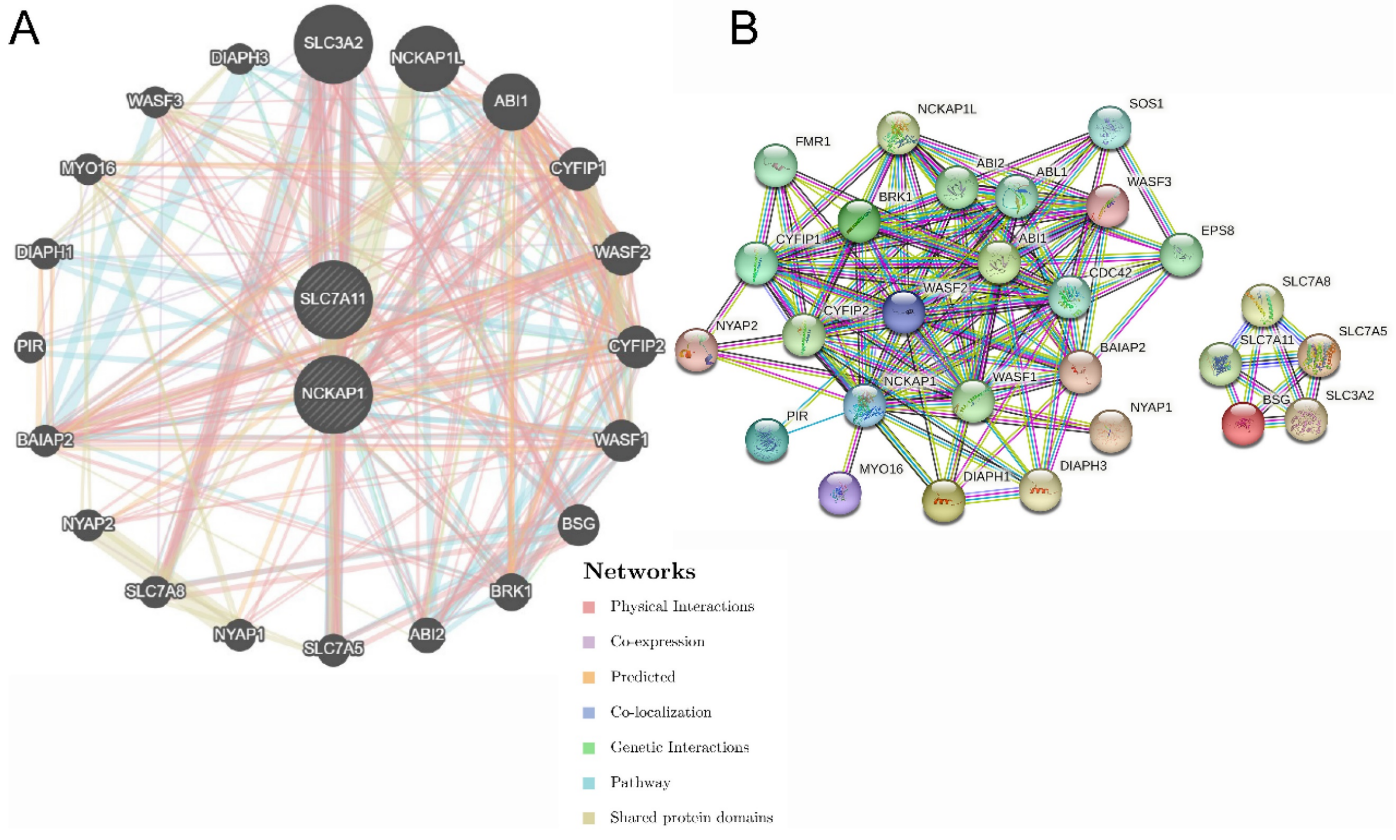
** The gene pairs and PPI network of SCL7A11 and NCKAP1.** A: The top 20 genes associated with NCKAP1 and SLC7A11 using GeneMANIA. B: The PPI network of interactions proteins with NCKAP1 and SLC7A11.

The functions of NCKAP1 and SLC7A11 were further explored. Twenty genes were significantly enriched by NCKAP1 and SLC7A11, and the network output is shown in Fig. 5A by GeneMANIA. All of the nodes were analysed using STRING, and the PPI network is shown in Fig. 5B in order to illustrate the protein-protein interaction relationship. Moreover, biological process, molecular function, cellular component, and KEGG pathway analyses were identified and visualized in Fig. S3 and Table S3. The biological and cellular functions were focused on actin activities, GTPase and immunoreaction.

### 3.6 Comprehensive evaluation of the immune landscape in GC

**Figure 6:**
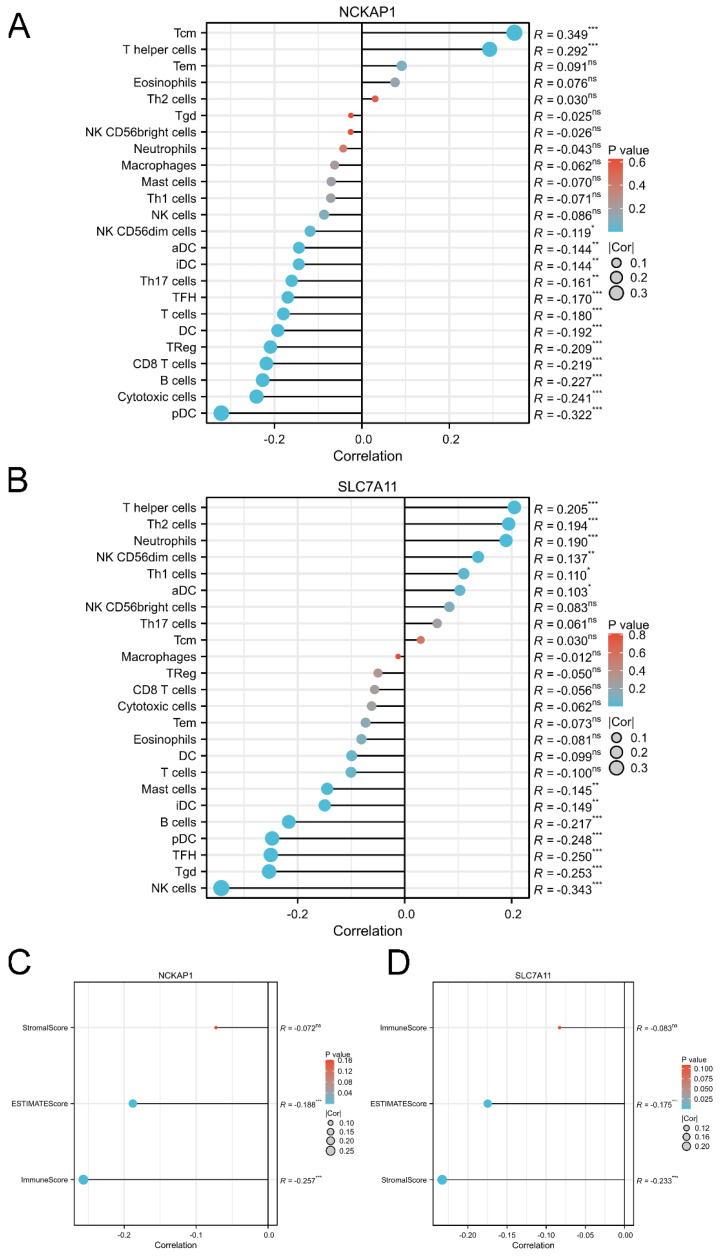
** The correlation between the expression level of NCKAP1, SLC7A11 and immune cell infiltration.** A: The lollipop diagram of the correlation between the expression of NCKAP1 and immune cell infiltration levels in TCGA cohort. B: The lollipop diagram of the correlation between the expression of SLC7A11 and immune cell infiltration levels in TCGA cohort. C: The stromal score, estimate score, immune score of different expression level of NCKAP1 of GC samples in TCGA cohort. D: The stromal score, estimate score, immune score of different expression level of SLC7A11 of GC samples in TCGA cohort.

Based on the immunoreaction of the functional analysis, we further described the immune landscape of NCKAP1 and SLC7A11. Our results showed that the expression of NCKAP1 was significantly associated with the infiltration of immune cells, such as T central memory (Tcm) cells, T helper cells, and plasmacytoid DCs, as shown in Fig. 6A. Furthermore, the expression level of SLC7A11 correlated with immune cell infiltration, including helper cells, Th2 cells, and neutrophils (Fig. 6B). Meanwhile, both NCKAP1 and SLC7A11 were related to the ESTIMATE score and immune score (Fig. 6C-D). Moreover, we systemically evaluated the relationships of both molecules and microenvironment, chemokines, chemokine receptors, immunoinhibitors, immunostimulators, MHC molecules, and target drugs, as shown in Fig. S4-5.

### 3.7 Establishment of the ceRNA regulatory network

**Figure 7:**
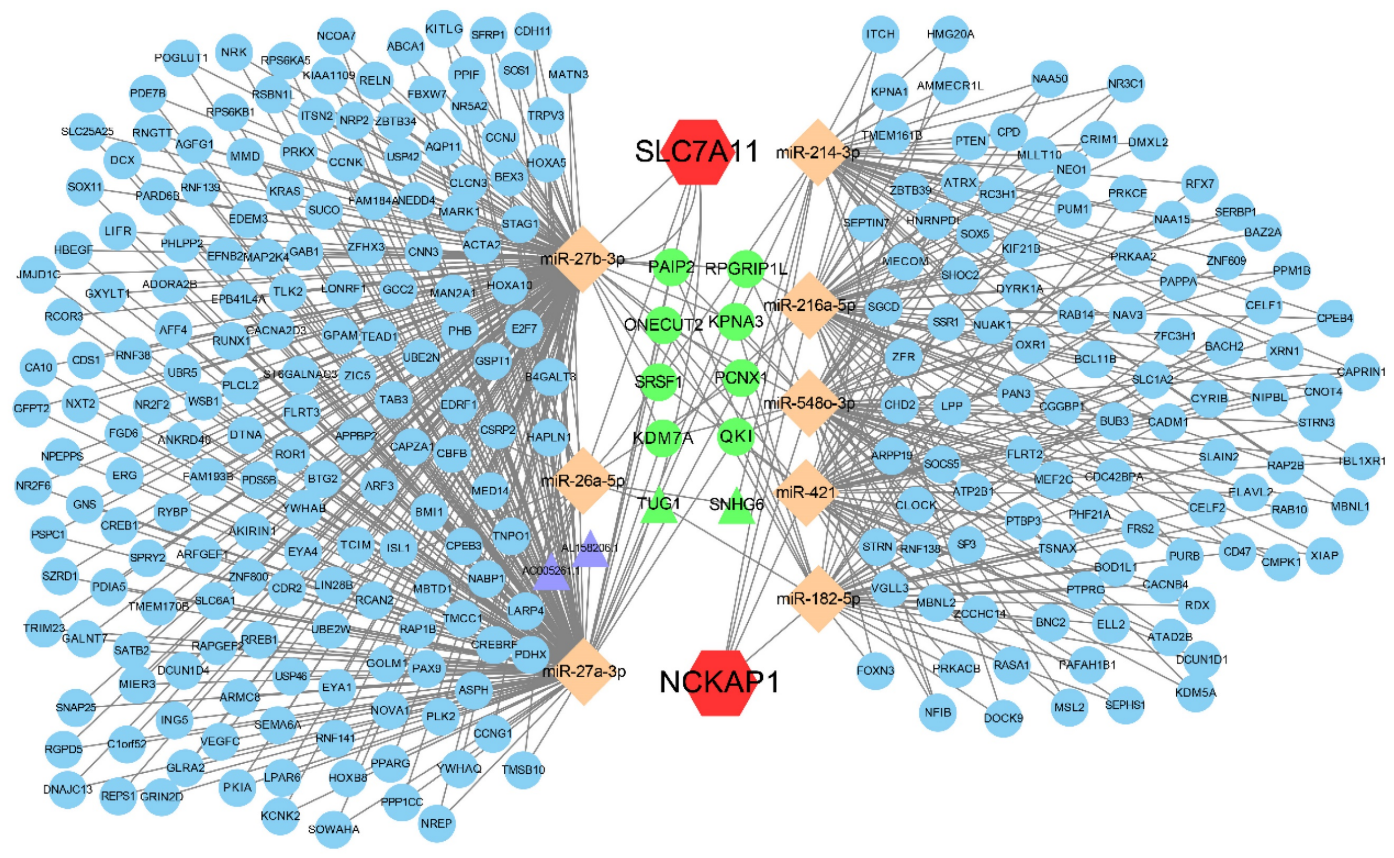
** The ceRNA regulatory network of NCKAP1 and SLC7A11.** Red hexagons represent NCKAP1 and SLC7A11, orange rhombuses represent the miRNAs, purple triangles represent the lncRNAs, blue circles represent the mRNAs and green represents the co-regulated molecules.

Aiming to reveal the downstream regulatory mechanisms of NCKAP1 and SLC7A11, the interactions of miRNAs and lncRNAs were investigated. Our result showed that NCKAP1 bound to 5 miRNAs and SLC7A11 bound to 3 miRNAs. More interestingly, TUG1 and SNHG6 (2 lncRNAs) as well as 8 mRNAs were co-regulated by NCKAP1 and SLC7A11 via miRNAs (Fig. 7), which provided important targets for further research. The expression of the co-regulated targets was detected via TCGA cohort shown in Fig. S6. In addition, the relationship between RNA binding proteins and NCKAP1, SLC7A11 was shown in Table S4. Finally, biological process, molecular function, cellular component, and KEGG pathway analyses displayed the potential function of the ceRNA in Fig. S7.

### 3.8 Validation of the differential expression and clinical significance of NCKAP1 and SLC7A11

**Figure 8:**
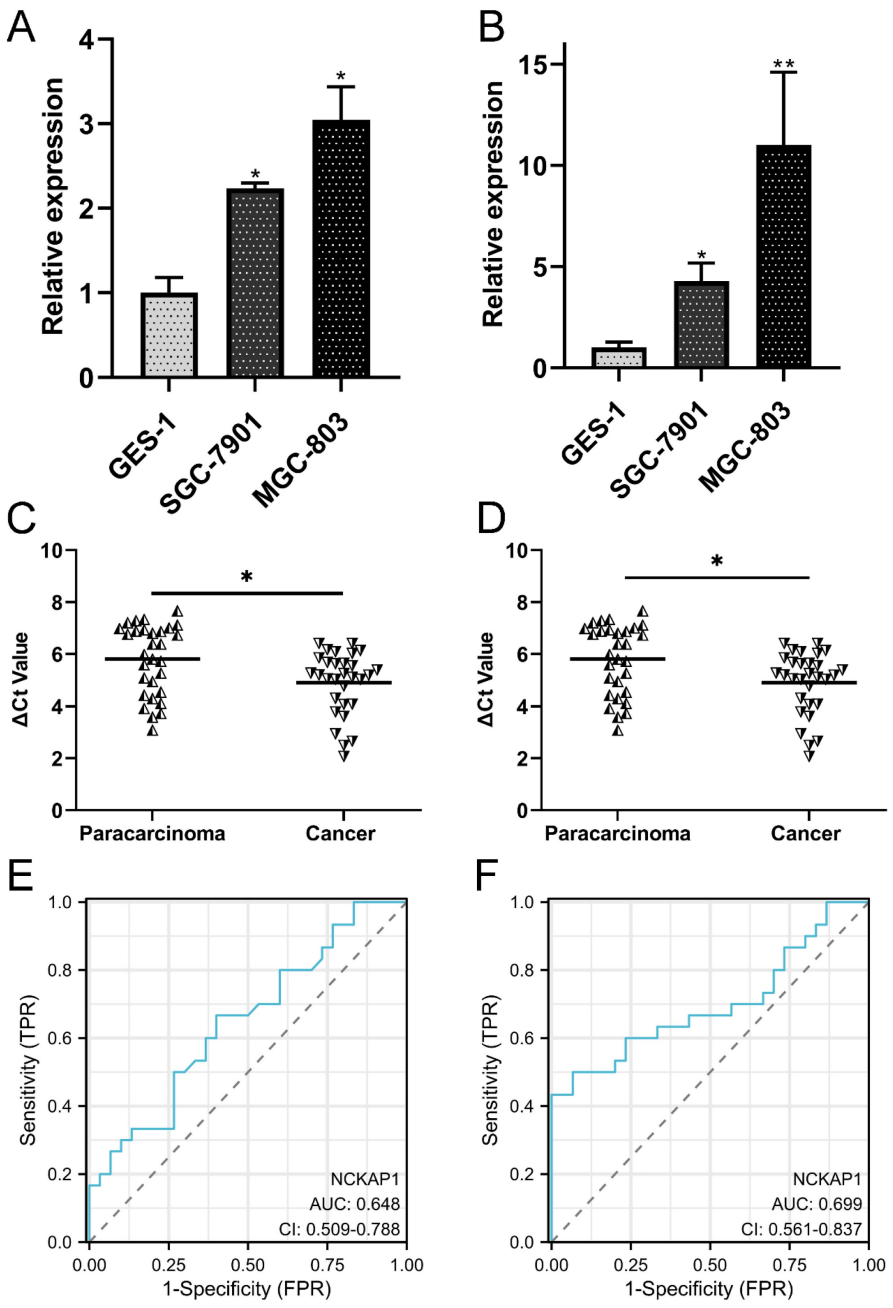
** Validation of the expression and diagnostic values of NCKAP1 and SLC7A11.** A: NCKAP1 was significantly upregulated in the GC cells. B: SLC7A11was significantly upregulated in the GC cells. C: NCKAP1 was significantly upregulated in the GC tissues compared to the paracarcinoma. D: SLC7A11 was significantly upregulated in the GC tissues compared to the paracarcinoma. E: The ROC curve of NCKAP1 in GC tissues and normal tissues. F: The ROC curve of SLC7A11 in GC tissues and normal tissues.

To validate the differential expression of both genes, qRT-PCR was performed to detect the expression levels of NCKAP1 and SLC7A11 in the cell line and tissue line. Our results showed that NCKAP1 and SLC7A11 were both upregulated in GC cells (Fig. 8A-B). The results of paired GC tissues showed that NCKAP1 and SLC7A11 were also overexpressed in GC tissues (Fig. 8C-D). The AUC, cut-off line, sensitivity, and specificity of the ROC curve of NCKAP1 were 0.648, 3.16, 66.7%, and 60%, respectively (Fig. 8E). The AUC, cut-off line, sensitivity, and specificity of the ROC curve of SLC7A11 were 0.699, 6.275, 50.0%, and 93.3%, respectively (Fig. 8F). All of these results suggested that higher NCKAP1 and SLC7A11 were promising prognostic and diagnostic biomarkers in GC.

## 4. Discussion

Currently, the diagnostic efficacy and prognosis of GC are still not ideal despite developments and breakthroughs in surgery, radiotherapy combined with chemotherapy and immunological regulators 31, 32. Moreover, the aetiology and pathogenesis of GC are multifactorial and poorly understood. Hence, it is important to identify novel tumour biomarkers and elucidate the molecular mechanisms of tumour initiation and progression.

Abnormal programmed cell death and apoptosis are critical pathways for tumour growth and development. Illustration of uncontrolled proliferation and apoptosis can facilitate recovery of the balance of the cell cycle, which is helpful to design sparking tumour biomarkers and immunotherapy targets. Disulfidptosis is a unique and novel type of cell death that is different from traditional apoptosis and necrosis and triggers cell death by promoting actin polymerization and lamellipodia formation, inducing aberrant accumulation of intracellular disulphides 33. NCKAP1, RPN1, SLC3A2, and SLC7A11 are the key genes in the progression of disulfidptosis, and their potential to lead to actin network collapse and cell death in GC is unknown. It has been demonstrated that loss of NCKAP1 can affect major actin nucleators in lamellipodia formation in fibroblasts by influencing spreading and focal adhesion dynamics, indicating the role of NCKAP1 in cell migration 34. Moreover, NCKAP1 significantly inhibited cell proliferation, invasion and migration in clear cell renal cell carcinoma and is a prognostic biomarker for clinical application 35. The expression level of SLC7A11 is regulated by stress, such as oxidative stress and genotoxic stress, which further induces cell death and apoptosis 36. However, the clinical value and cell function of these genes in GC remain unclear. In this study, we found that NCKAP1 and SLC7A11 were independent risk factors for GC survival time and established a prognostic nomogram. The validity of the model was proven by a calibration plot and DCA curves. Moreover, we further explored and validated their values towards diagnostic application. The analysis of TCGA samples and clinical samples displayed satisfactory results for the AUC, sensitivity and specificity of NCKAP1 and SLC7A11 overexpression for GC screening. All of the results implied that NCKAP1 and SLC7A11 were potential prognostic and diagnostic biomarkers for GC and are worthy of a larger, multicentre randomized clinical trial. In addition, we revealed the integrated functions of NCKAP1, SLC7A11 and associated genes in a functional network and explored their possible functions via enrichment analysis. These genes and proteins may influence GC development by regulating the activities of actin, energy metabolism of GTPase and immunoreaction, which is closely related to the process of disulfidptosis, as mentioned before.

Special immune-related genes can reflect the GC immune microenvironment and predict the efficacy of immune checkpoint inhibitors therapy 37. In fact, numerous studies have linked high mutation burdens of tumor with immunotherapy responses, and immunotherapy strategies have made progress 38-41. For instance, chemotherapy and pembrolizumab plus trastuzumab display obvious benefits of improving overall survival time in GC patients and are approved as first-line treatments for Her2-positive GC 42. Nivolumab is a monoclonal antibody inhibitor of PD-1 that has been indicated to provide durable responses with manageable safety in patients with advanced GC who progressed following second-line treatment 43. However, the exact contribution and durable responses of immunotherapy are still uncertain, and it is necessary to assess the objective response rate as well as novel treatment targets 44. In this work, we found the relationship of the MMR genes and the expression of NCKAP1 and SLC7A11, which was essential to anti-tumor immunity 45. the Meanwhile, overexpression of NCKAP1 and SLC7A11 was simultaneously associated with the infiltration of T helper cells, NK CD56dim cells, activated DCs (aDCs), immature DCs (iDCs), T follicular helper cells (TFHs), B cells, and plasmacytoid DCs (pDCs). Subsequently, the expression of SLC7A11 was associated with several drugs, such as riluzole and sulfasalazine, to regulate downstream genes, which provided useful information and directions for future clinical research 46. Subsequently, we systematically delineate an overall immune landscape related to treatment, which provides promising immune treatment targets with the ultimate goal of improving clinical outcomes and survivorship.

With the emerging appreciation for the significance of ncRNAs, the present studies pay attention to determine the roles of ceRNA in the process of participating tumor initiation and progress 47. miRNAs play important roles in cancer-related immune regulation whose expression correlates with tumor mutation burden and immune regulation 48. Meanwhile, lncRNAs can function as competing endogenous RNAs to impair the miRNA inhibition on targeted mRNAs, further regulating gene expression, protein translation and malignant biological properties 49. Recent study has been demonstrated that disulfidptosis-associated lncRNAs have potential to predict the prognosis, tumor microenvironment, and immunotherapy and chemotherapy options in colon adenocarcinoma, which strongly implies the significance of the correlation between lncRNA and disulfidptosis-associated genes 50. Our ceRNA network showed the interactions of co-regulated lncRNAs and mRNAs of NCKAP1 and SLC7A11, which points out the direction of future research of downstream regulation signal pathway.

The limitations of our present study were the lack of functional verification results, which will be assessed in future studies.

## 5. Conclusion

In conclusion, NCKAP1 and SLC7A11 are promising prognostic and diagnostic biomarkers for GC, which correlate with the activities of actin, energy metabolism of GTPase, immune infiltration and immunotherapy.

## Supplementary Material

Supplementary figures and tables.

## Figures and Tables

**Table 1:** Univariate and multivariate regression of clinical characteristics.

Characteristics	Total(N)	Univariate analysis			Multivariate analysis	
		Hazard ratio (95% CI)	P value		Hazard ratio (95% CI)	P value
NCKAP1	370	1.196 (0.917 - 1.560)	0.187		1.455 (1.075 - 1.969)	**0.015**
SLC3A2	370	0.868 (0.699 - 1.078)	0.200			
SLC7A11	370	0.915 (0.808 - 1.036)	0.162		0.776 (0.667 - 0.904)	**0.001**
Age	367	1.022 (1.005 - 1.039)	**0.009**		1.040 (1.021 - 1.060)	**< 0.001**
Gender	370		0.182			
Male	237	Reference			Reference	
Female	133	0.789 (0.554 - 1.123)	0.188		0.626 (0.425 - 0.922)	**0.018**
Pathologic T stage	362		**0.003**			
T1	18	Reference			Reference	
T2	78	6.725 (0.913 - 49.524)	0.061		4.341 (0.578 - 32.585)	0.153
T3	167	9.548 (1.326 - 68.748)	**0.025**		5.995 (0.814 - 44.152)	**0.079**
T4	99	9.634 (1.323 - 70.151)	**0.025**		6.048 (0.803 - 45.564)	**0.081**
Pathologic N stage	352		**0.001**			
N0	107	Reference			Reference	
N1&N2&N3	245	1.925 (1.264 - 2.931)	**0.002**		1.680 (1.064 - 2.654)	**0.026**
Pathologic M stage	352		**0.010**			
M0	327	Reference			Reference	
M1	25	2.254 (1.295 - 3.924)	**0.004**		2.466 (1.307 - 4.652)	**0.005**
Histologic grade	361		0.169			
G1	10	Reference			Reference	
G2	134	1.648 (0.400 - 6.787)	0.489		1.980 (0.269 - 14.579)	0.503
G3	217	2.174 (0.535 - 8.832)	0.278		2.510 (0.344 - 18.326)	0.364

Entry: 0.05; Removal: 0.10, *P* <0.05
