# Supplementary material for: Clinical Significance of Disulfidptosis-related Genes and Functional Analysis in Gastric Cancer
Source: J Cancer. 2024 Jan 1;15(4):1053–66. doi: 10.7150/jca.91796 (PMC10788733; doi:10.7150/jca.91796)
Supplement: Supplementary file 1 — Supplementary figures and tables. [file jcav15p1053s1.pdf]

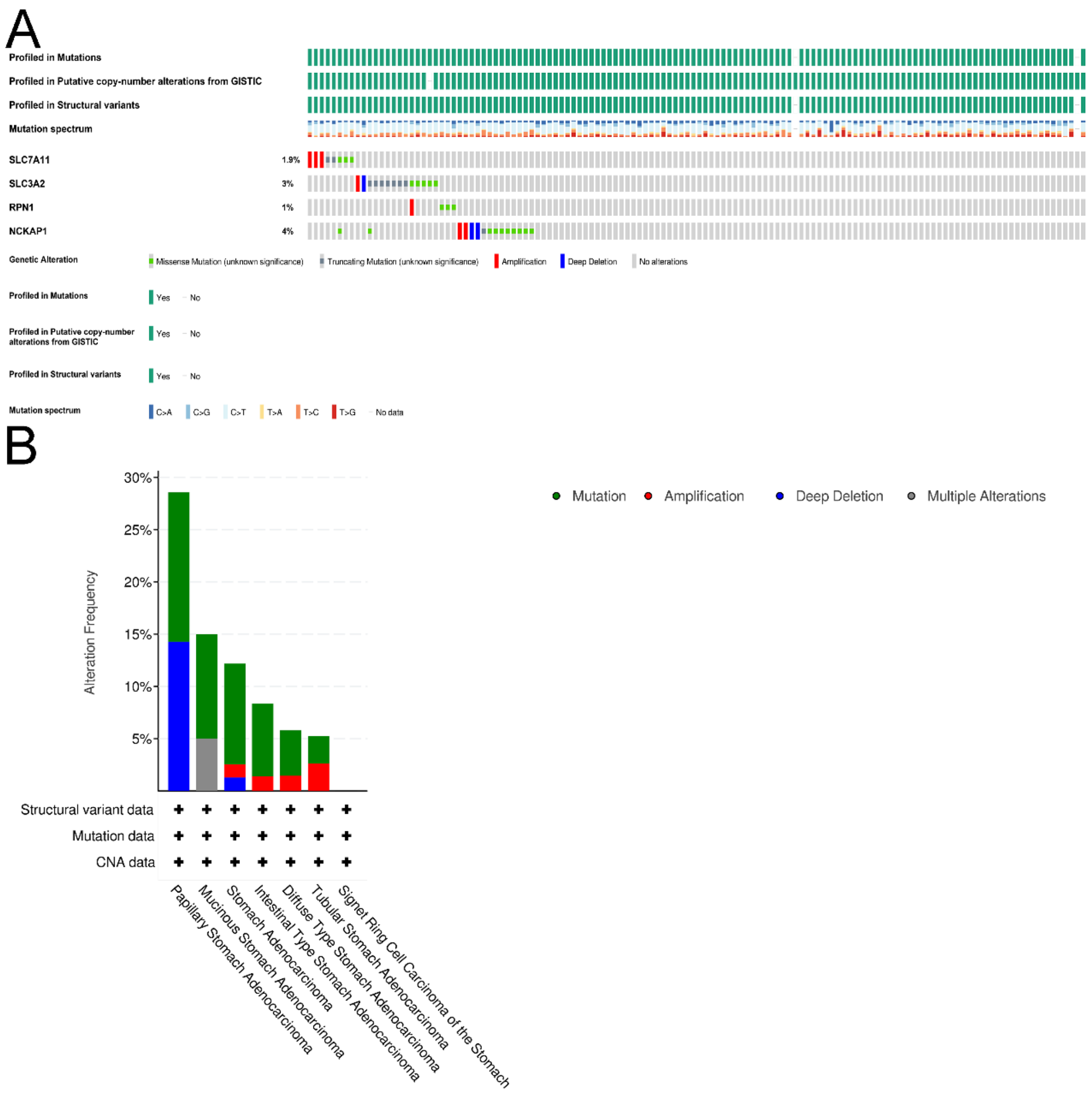
A
Profiled in Mutations
Profiled in Putative copy-number alterations from GISTIC
Profiled in Structural variants
Mutation spectrum
SLC7A11
1.9%
SLC3A2
3%
RPN1
1%
NCKAP1
4%
Genetic Alteration
Missense Mutation (unknown significance)
Truncating Mutation (unknown significance)
Amplification
Deep Deletion
No alterations
Profiled in Mutations
Yes
No
Profiled in Putative copy-number alterations from GISTIC
Yes
No
Profiled in Structural variants
Yes
No
Mutation spectrum
C>A
C>G
C>T
T>A
T>C
T>G
No data
B
Mutation
Amplification
Deep Deletion
Multiple Alterations
30%
25%
20%
15%
10%
5%
Alteration Frequency
Structural variant data
Mutation data
CNA data
Papillary Stomach Adenocarcinoma
Mucinous Stomach Adenocarcinoma
Stomach Adenocarcinoma
Intestinal Type Stomach Adenocarcinoma
Diffuse Type Stomach Adenocarcinoma
Tubular Stomach Adenocarcinoma
Signet Ring Cell Carcinoma of the Stomach

Sup Fig. 1

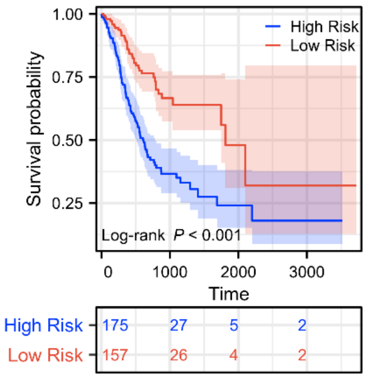

High Risk
Low Risk
Survival probability
1.00
0.75
0.50
0.25
Log-rank P < 0.001
0
1000
2000
3000
Time
High Risk 175 27 5 2
Low Risk 157 26 4 2

Sup Fig. 2

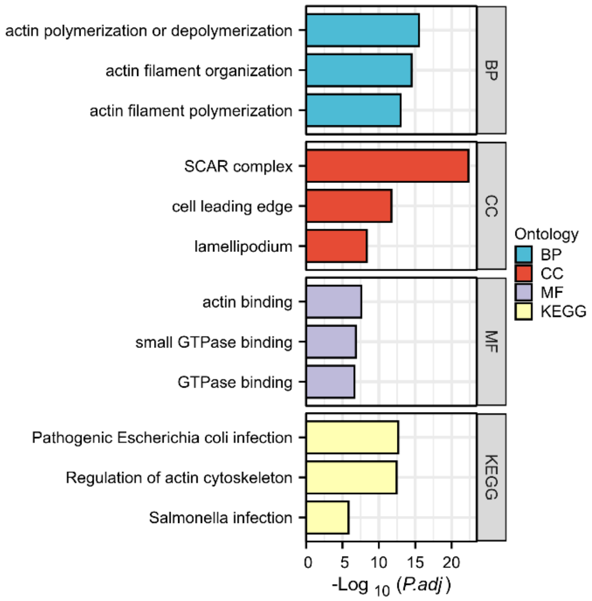
actin polymerization or depolymerization
actin filament organization
actin filament polymerization
SCAR complex
cell leading edge
lamellipodium
actin binding
small GTPase binding
GTPase binding
Pathogenic Escherichia coli infection
Regulation of actin cytoskeleton
Salmonella infection
BP
CC
MF
KEGG
0
5
10
15
20
-Log 10 (P.adj)
Ontology
BP
CC
MF
KEGG

Sup Fig. 3

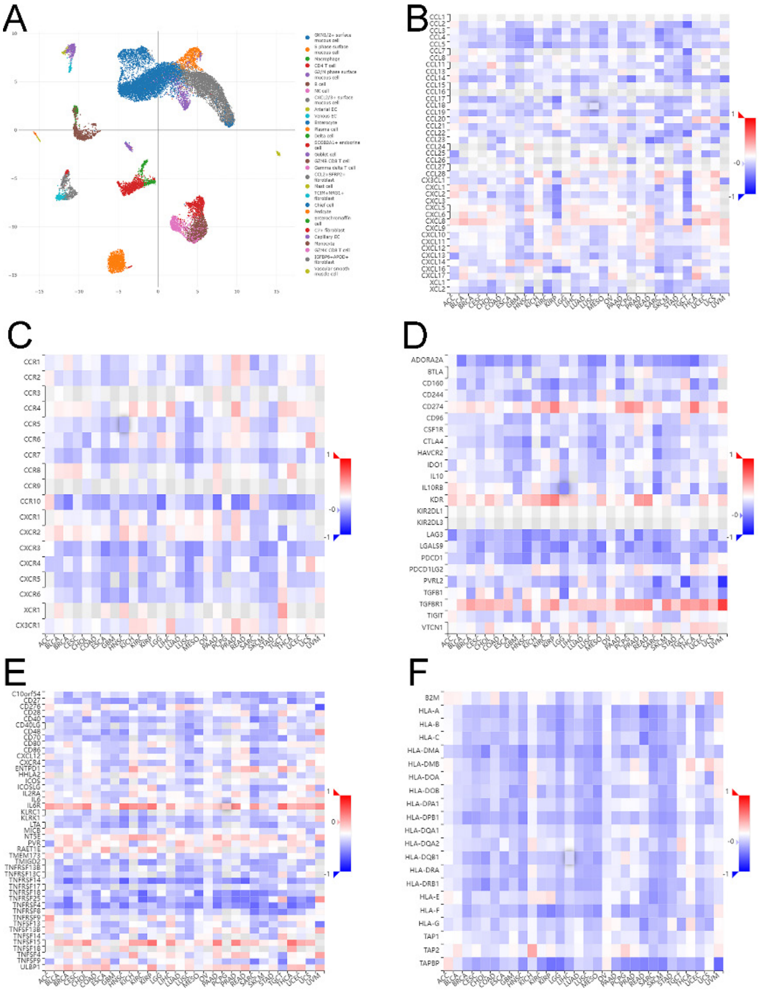
A
B
C
D
E
F
CCR1
CCR2
CCR3
CCR4
CCR5
CCR6
CCR7
CCR8
CCR9
CCR10
CXCR1
CXCR2
CXCR3
CXCR4
CXCR5
CXCR6
XCR1
CX3CR1
ADORA2A
BTLA
CD160
CD244
CD274
CD96
CSF1R
CTLA4
HAVCR2
IDO1
IL10
IL10RB
KDR
KIR2DL1
KIR2DL3
LAG3
LGALS9
PDCD1
PDCD1LG2
PVRL2
TGFB1
TGFBR1
TIGIT
VTCN1
B2M
HLA-A
HLA-B
HLA-C
HLA-DMA
HLA-DMB
HLA-DOA
HLA-DOB
HLA-DPA1
HLA-DPB1
HLA-DQA1
HLA-DQA2
HLA-DQB1
HLA-DRA
HLA-DRB1
HLA-E
HLA-F
HLA-G
TAP1
TAP2
TAPBP

Sup Fig. 4

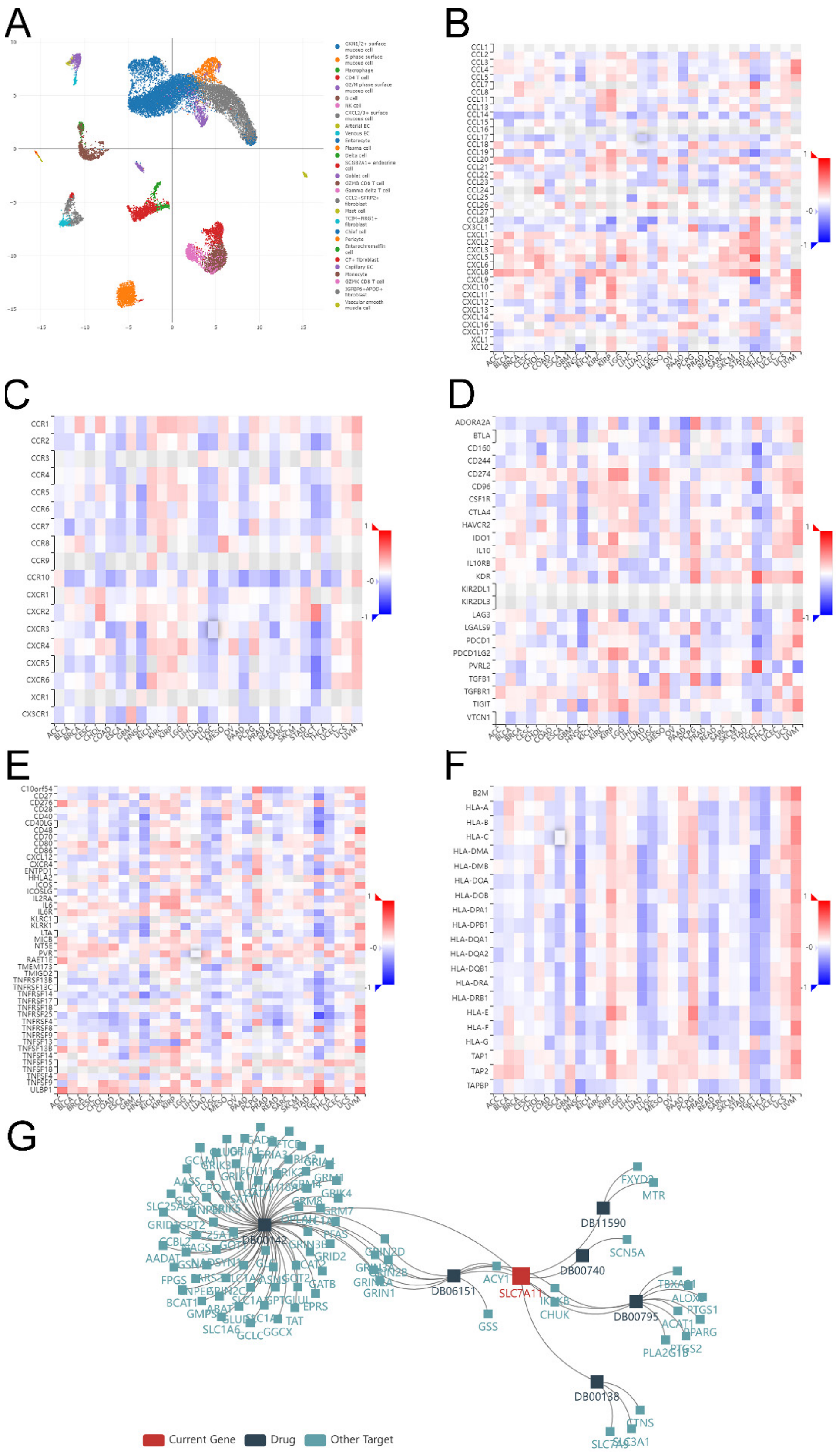
A
B
C
D
E
F
G
CCL1 CCL2 CCL3 CCL4 CCL5 CCL7 CCL8 CCL11 CCL13 CCL14 CCL15 CCL16 CCL17 CCL18 CCL19 CCL20 CCL21 CCL22 CCL23 CCL24 CCL25 CCL26 CCL27 CCL28 CX3CL1 CXCL1 CXCL2 CXCL3 CXCL5 CXCL6 CXCL8 CXCL9 CXCL10 CXCL11 CXCL12 CXCL13 CXCL14 CXCL16 CXCL17 XCL1 XCL2
CCR1 CCR2 CCR3 CCR4 CCR5 CCR6 CCR7 CCR8 CCR9 CCR10 CXCR1 CXCR2 CXCR3 CXCR4 CXCR5 CXCR6 XCR1 CX3CR1
ADORA2A BTLA CD160 CD244 CD274 CD96 CSF1R CTLA4 HAVCR2 IDO1 IL10 IL10RB KDR KIR2DL1 KIR2DL3 LAG3 LGALS9 PDCD1 PDCD1LG2 PVRL2 TGFB1 TGFBR1 TIGIT VTCN1
B2M HLA-A HLA-B HLA-C HLA-DMA HLA-DMB HLA-DOA HLA-DOB HLA-DPA1 HLA-DPB1 HLA-DQA1 HLA-DQA2 HLA-DQB1 HLA-DRA HLA-DRB1 HLA-E HLA-F HLA-G TAP1 TAP2 TAPBP
ACC BLCA BRCA CESC CHOL COAD DLBC ESCA GBM HNSC KICH KIRC KIRP LGG LIHC LUAD LUSC MESO OV PAAD PCPG PRAD READ SARC SKCM STAD TGCT THCA THYM UCEC UCS UVM
DB00142 DB06151 DB11590 DB00740 DB00795 DB00138
SLC7A11
Current Gene
Drug
Other Target

Sup Fig. 5

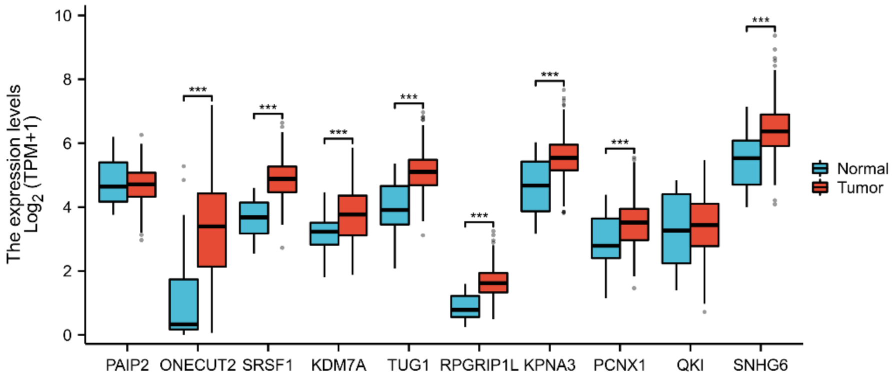

10
8
6
4
2
0
The expression levels
Log_2 (TPM+1)
***
PAIP2
ONECUT2
SRSF1
KDM7A
TUG1
RPGRIP1L
KPNA3
PCNX1
QKI
SNHG6
Normal
Tumor

Sup Fig. 6

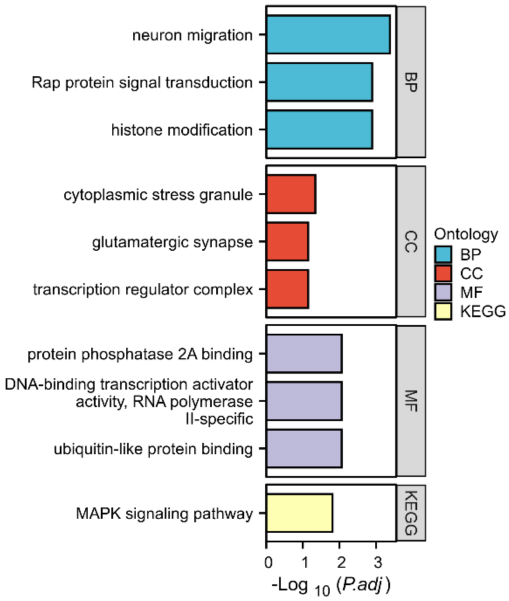
neuron migration
Rap protein signal transduction
histone modification
cytoplasmic stress granule
glutamatergic synapse
transcription regulator complex
protein phosphatase 2A binding
DNA-binding transcription activator activity, RNA polymerase II-specific
ubiquitin-like protein binding
MAPK signaling pathway
BP
CC
MF
KEGG
Ontology
BP
CC
MF
KEGG
0
1
2
3
-Log 10 (P.adj)

Sup Fig. 7

Supplementary Table 1 Correlation between NCKAP1 expression levels in gastric cancer tissues and clinicopathological parameters in TCGA cohort

Characteristics	Low expression of NCKAP1	High expression of NCKAP1	P value
n (%)	187	188	
Age, median (IQR)	67 (57, 73)	68 (59, 74)	0.336
Gender,			0.493
Male	117 (31.2%)	124 (33.1%)	
Female	70 (18.7%)	64 (17.1%)	
Pathologic T stage			0.450
T1	10 (2.7%)	9 (2.5%)	
T2	41 (11.2%)	39 (10.6%)	
T3	91 (24.8%)	77 (21%)	
T4	44 (12%)	56 (15.3%)	
Pathologic N stage			0.921
N0	59 (16.5%)	52 (14.6%)	
N1	49 (13.7%)	48 (13.4%)	
N2	36 (10.1%)	39 (10.9%)	
N3	38 (10.6%)	36 (10.1%)	
Pathologic M stage			0.293
M0	162 (45.6%)	168 (47.3%)	
M1	15 (4.2%)	10 (2.8%)	
Pathologic stage			0.391
Stage I	24 (6.8%)	29 (8.2%)	
Stage II	64 (18.2%)	47 (13.4%)	
Stage III	73 (20.7%)	77 (21.9%)	
Stage IV	20 (5.7%)	18 (5.1%)	

Characteristics	Low expression of NCKAP1	High expression of NCKAP1	P value
Primary therapy outcome			0.176
PD	27 (8.5%)	38 (12%)	
SD&PR	12 (3.8%)	9 (2.8%)	
CR	125 (39.4%)	106 (33.4%)	

Supplementary Table 2 Correlation between SLC7A11 expression levels in gastric cancer tissues and clinicopathological parameters in TCGA cohort

Characteristics	Low expression of SLC7A11	High expression of SLC7A11	P value
n (%)	187	188	
Age, median (IQR)	64 (57.25, 72)	69 (59, 75)	0.004
Gender			0.299
Male	125 (33.3%)	116 (30.9%)	
Female	62 (16.5%)	72 (19.2%)	
Pathologic T stage			0.092
T1	8 (2.2%)	11 (3%)	
T2	44 (12%)	36 (9.8%)	
T3	91 (24.8%)	77 (21%)	
T4	40 (10.9%)	60 (16.3%)	
Pathologic N stage			0.763
N0	56 (15.7%)	55 (15.4%)	
N1	49 (13.7%)	48 (13.4%)	
N2	37 (10.4%)	38 (10.6%)	
N3	32 (9%)	42 (11.8%)	
Pathologic M stage			0.159
M0	167 (47%)	163 (45.9%)	
M1	9 (2.5%)	16 (4.5%)	
Pathologic stage			0.037
Stage I	24 (6.8%)	29 (8.2%)	
Stage II	66 (18.8%)	45 (12.8%)	
Stage III	72 (20.5%)	78 (22.2%)	
Stage IV	13 (3.7%)	25 (7.1%)	

Characteristics	Low expression of SLC7A11	High expression of SLC7A11	P value
Primary therapy outcome			0.589
PD	32 (10.1%)	33 (10.4%)	
SD&PR	13 (4.1%)	8 (2.5%)	
CR	118 (37.2%)	113 (35.6%)	

Supplementary Table 3 Go analysis and KEGG analysis

Ontology	ID	Description	GeneRatio	BgRatio	pvalue	p.adjust
BP	GO:0008154	actin polymerization or depolymerization	13/27	199/18800	2.48e-19	3.02e-16
BP	GO:0007015	actin filament organization	15/27	450/18800	5.15e-18	3.13e-15
BP	GO:0030041	actin filament polymerization	11/27	169/18800	2.57e-16	1.04e-13
BP	GO:0032956	regulation of actin cytoskeleton organization	13/27	352/18800	4.42e-16	1.34e-13
BP	GO:0110053	regulation of actin filament organization	12/27	270/18800	8.66e-16	2.05e-13
CC	GO:0031209	SCAR complex	9/25	12/19594	3.83e-25	4.94e-23
CC	GO:0031252	cell leading edge	12/25	416/19594	2.9e-14	1.87e-12
CC	GO:0030027	lamellipodium	8/25	202/19594	1.03e-10	4.44e-09
CC	GO:0098858	actin-based cell projection	8/25	217/19594	1.83e-10	5.9e-09
CC	GO:0001726	ruffle	7/25	177/19594	1.83e-09	4.71e-08
MF	GO:0003779	actin binding	10/26	439/18410	2.03e-10	2.68e-08
MF	GO:0031267	small GTPase binding	8/26	267/18410	2.2e-09	1.45e-07
MF	GO:0051020	GTPase binding	8/26	298/18410	5.2e-09	2.29e-07
MF	GO:0015175	neutral amino acid transmembrane transporter activity	4/26	41/18410	3.05e-07	1.01e-05
MF	GO:0071933	Arp2/3 complex binding	3/26	15/18410	1.13e-06	2.97e-05

Ontology	ID	Description	GeneRatio	BgRatio	pvalue	p.adjust
KEGG	hsa05130	Pathogenic Escherichia coli infection	12/20	197/8164	2.97e-15	2.11e-13
KEGG	hsa04810	Regulation of actin cytoskeleton	12/20	218/8164	1.02e-14	3.6e-13
KEGG	hsa05132	Salmonella infection	8/20	249/8164	6.14e-08	1.45e-06
KEGG	hsa04520	Adherens junction	5/20	71/8164	6.04e-07	1.07e-05
KEGG	hsa04666	Fc gamma R-mediated phagocytosis	4/20	97/8164	7.84e-05	0.0010

Supplementary Table 4 Correlation between RNA binding proteins and NCKAP1, SLC7A11

**Co-RNA binding proteins**	NCKAP1	SLC7A11
U2AF2	RNPS1	ELAVL1
SCAF8	U2AF2	RBM47
YBX1	RBM10	U2AF1
SCAF4	SCAF8	SND1
BCLAF1	YBX1	SCAF4
PTBP1	SCAF4	EIF4A3
IGF2BP2	RBMX	U2AF2
GRWD1	FXR1	HNRNPA2B1
EIF4A3	BCLAF1	IGF2BP2
SRSF1	PTBP1	MTDH
U2AF1	FXR1	IGF2BP2
SND1	IGF2BP2	IGF2BP3
IGF2BP3	GRWD1	SRSF1
	TARDBP	BCLAF1
	ALYREF	SCAF8
	YTHDF3	YBX1
	CPSF7	GRWD1
	FUS	RBFOX2
	EIF4A3	PTBP1
	DDX54	PRPF8
	SRSF1	IGF2BP3
	ZC3H7B	GRWD1
	SRSF7	DAP3
	U2AF1	BCLAF1

**Co-RNA binding proteins**	NCKAP1	SLC7A11
	IGF2BP1	AUH
	FUS	YBX1
	DDX54	
	RC3H1	
	NUDT21	
	MBNL2	
	TRA2B	
	SRRM4	
	SND1	
	G3BP1	
	HNRNPC	
	RC3H1	
	IGF2BP3	
	LIN28A	
	LIN28B	
	NUDT21	
	G3BP1	
	IGF2BP3	
	HNRNPC	
	RC3H1	
	NUDT21	
